# Point-of-Care for Evaluating Antimicrobial Resistance through the Adoption of Functional Materials

**DOI:** 10.1021/acs.analchem.1c03856

**Published:** 2021-11-22

**Authors:** Sima Singh, Arshid Numan, Stefano Cinti

**Affiliations:** † IES Institute of Pharmacy, IES University Campus, Kalkheda, Ratibad Main Road, Bhopal 462044, Madhya Pradesh, India; ‡ Graphene & Advanced 2D Materials Research Group (GAMRG), School of Engineering and Technology, Sunway University, 5, Jalan University, Bandar Sunway, 47500 Petaling Jaya, Selangor, Malaysia; § Department of Pharmacy, University of Naples “Federico II”, Via D. Montesano 49, 80131 Naples, Italy; ∥ BAT Center−Interuniversity Center for Studies on Bioinspired Agro-Environmental Technology, University of Napoli Federico II, 80055 Naples, Italy

Modern medicine has adopted the term “antimicrobials”, favoring the term “antibiotic” to emphasize the inclusion of antiviral, antifungal, and antiparasitic medications. However, both words are interchangeable.^1^ Antimicrobial is a tiny molecule that can inhibit, kill, or prevent the development of microbes. Specific terms, such as antibacterial and antifungal, will be used where they are suitable. While these tiny compounds are frequently employed to treat bacterial infections, certain bacteria can grow and survive in the face of antimicrobial pressures, a phenomenon known as antimicrobial resistance (AMR).^2^ Over the course of the previous 60 years, millions of metric tons of antibiotics have been manufactured and used for various medical conditions.^3^ The statistics indicate that antibiotics and antifungals have revolutionized both medicine and agriculture. It appears that a turning point has been reached in which antimicrobial drugs have been misused to create a global AMR issue. AMR development leads to medication inefficiency and chronic infections, increasing the risk of severe illness and transmission.^4^

Bacterial infections caused by “superbugs” are increasing globally, and conventional antibiotics are becoming less effective against these bacteria, such that we risk entering a postantibiotic era. The parliamentary health and social care committee of the United Kingdom has issued a dire warning about antimicrobial resistance. In essence, we are being warned that modern medicine will cease to exist unless something is done to combat this threat by quoting, “Quite simply, if action is not taken to address this growing threat, we are told that modern medicine will be lost”. An estimated 10 million people per year will be killed each year due to AMR, which is greater than the number of people killed by cancer and diabetes combined and will result in a 2 to 3.5% decrease in the gross domestic product (GDP). It might cost the world upward of 100 trillion dollars USD.^5^ Though they are predicted to be unclear and depressing, the current forecasts are now accepted throughout the scientific community. The 20-year vision and 5-year plan (2019–2024) for tackling AMR in the United Kingdom encompass humans, animals, food, and the environment, with collaboration and transdisciplinary approaches at the local, regional, national, and international levels to achieve optimal health outcomes.^6^

AMR is unquestionably one of the most significant issues of our era. AMR has a substantial clinical and public health impact, which is expected to rise in the future. In order to provide better trustworthy, comprehensive, and actionable findings, these uncertainties need to be addressed. As a result, immediate action is required to address this issue. To tackle and address the severe threat of AMR basically, based on a 2-fold approach (1) to delay the development of AMR by wiser and more innovative usage of antimicrobial drugs and (2) to speed up new antimicrobial development.^7,8^ Effective antimicrobial treatment and AMR are intimately connected and helpful to make an accurate diagnosis.

Polymerase chain reaction (PCR) based detection is a “gold standard” technique due to high sensitivity and selectivity. While results from urine analysis are typically returned within 1 day, fecal or skin cultures may demand 2 days, and blood culture negatives are not considered conclusive until a 5-day incubation. Then they may still need further assessment in complementary media. Traditional microbial detection methods tend to be labor-intensive, expensive, and unportable^9^ due to limited access to diagnostic services and the inadequacy of existing testing. To properly monitor treatment therapy and identify AMR, there is an urgent need for rapid and straightforward detection techniques focused on on-site microbiological examination and to have an adequate degree of sensitivity and specificity.^10,11^ Additionally, AMR detection needs laboratories and clinics, which adds to the expense of treatments.

To close the gap between treatment and diagnosis, the World Health Organization (WHO) published a Global Action Plan on AMR in 2015. The plan emphasized the importance and need of “effective, fast, and low-cost diagnostic technologies for guiding the appropriate use of antibiotics in human and animal medicine.” This action plan aims to improve the speed and accuracy of diagnosis through quick, accurate, and minimal cost point-of-care testing (POCT) diagnostic technologies.^12^ POC-based diagnostics can correctly detect AMR pathogens at the bedside with a high detection rate, allowing for the rapid initiation of appropriate therapy while avoiding antibiotic overuse or abuse.

POC devices can alleviate some of the difficulties associated with traditional methods of detection. The POC methods provide AMR testing at the bedside or physician’s office by utilizing a urine specimen, blood, or oral fluid. It delivers disease-specific, portable, and easy to use without or with a little training based detection.^13^ The advantage of POCT over standard test procedures is shown in Figure 1.

**Figure 1:**
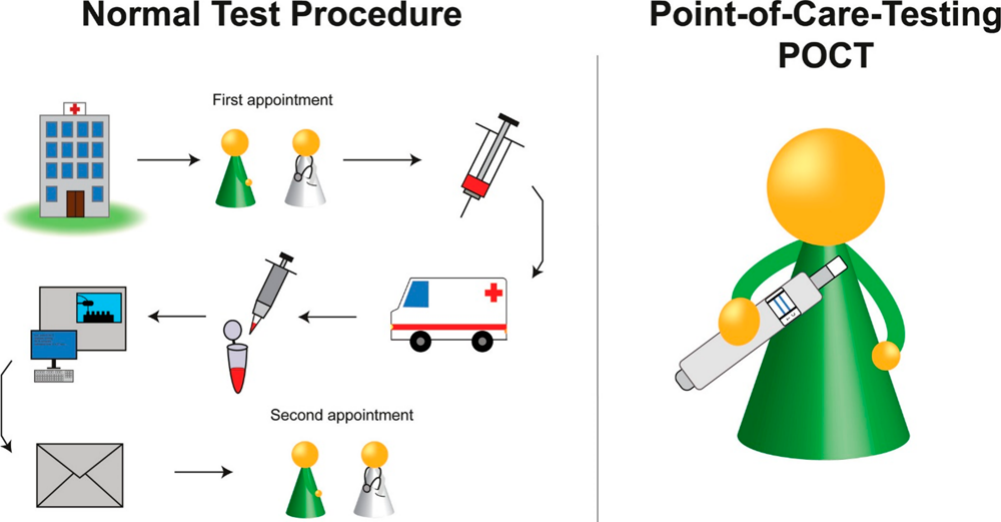
Difference between the conventional test procedure vs POCT. Adapted under the terms and conditions of the CC-BY license from Miesler, T.; Wimschneider, C.; Brem, A.; Meinel, L. *ACS Biomater. Sci. Eng.***2020**, *6* (5), 2709–2725 (ref (14)).

For sensitive and selective POC detection of AMR, nanomaterials combined with optical and electrochemical sensing platforms can meet the requirement for affordable, robust, and sensitive biosensors. Developing innovative, small, and cost-effective POCTs capable of providing specified output characteristics would broaden clinical applications and improve treatment results. To this end, the development of POCT and diagnostic tools for detecting AMR is expected to be a fruitful research domain in the near-decade. In this review, we revisit the fundamentals of POC sensors for AMR and describe the applications of nanomaterials in diagnosis, monitoring, and potential for utilizing these tools to improve primary care settings significantly. We offer a road plan with particular emphasis on opportunities for the future of precision medicine in AMR.

## Current Diagnostic Landscape and Its Limitations for AMR

Detecting AMRs present in bacteria is a crucial initial step in ensuring the administration of appropriate antibiotics to treat different infections. While highly established, time-efficient AMR detection technology is available, older traditional approaches are still in use. A wide range of screening procedures is presently accessible inside the healthcare system. This can be done through the use of growth-based (phenotypic) or molecular-based (genotypic) techniques. Some are frequently used in diagnostic laboratories, while others are still unutilized as research tools by academics and experts at various phases of development.^15^

Phenotypic assays can be employed in routine laboratory practice to detect the existence of acquired resistance mechanisms in nosocomial infections that are commonly isolated from hospitals and other healthcare facilities.^16^ Phenotypic techniques are mostly comprised of culture-based or staining-based diagnostic procedures. Currently, blood culture is the accepted method for the diagnosis of AMR. Microorganisms present in the blood are used to confirm the presence of these organisms. They have the benefits of being inexpensive, simple to conduct (automated systems), and having easily accessible interpretation criteria for frequently encountered species. Standard identification techniques have some drawbacks, including that findings might take up to 48 h (or more) to appear.^17^ Although this approach is highly informative, it is time-consuming, sometimes taking several days to run the entire panel of MICs on isolates after purification. Blood cultures and staining do not give enough information to guide antimicrobial treatment decisions.^18^

Molecular techniques can provide a faster and more reliable investigation of AMR than traditional phenotypic methods in many ways. These molecular diagnostic techniques help speed up bacterial detection/identification. However, they often involve specialized equipment and demand professional interpretation.^19,20^ Most genotypic methods entail an initial step in which the nucleic acid of interest is amplified. PCR, DNA microarray, whole-genome sequencing, and metagenomics are examples of such practices.^21^ Among molecular diagnostic techniques, PCR is a highly recognized detection tool.

On the other hand, molecular tests for identifying antimicrobial genes resistance and their genetic support are still under investigation.^22^ Traditional PCR techniques were eventually superseded by real-time PCR techniques (RT-PCR). For the microbiology laboratory, RT-PCR enables the development of regular diagnostic and therapeutic applications. Numerous studies have demonstrated how these approaches may be used to identify resistance determinants and monitor antimicrobial-resistant microorganisms.^23,24^ A downside to this approach is that novel resistance mechanisms may go undetected. In certain circumstances, the cost of developing an assay is too expensive, making this method unworkable. At the moment, the costs of equipment and reagents for PCR are too high for everyday use. Additionally, many laboratories struggle with appropriate quality control for molecular tests, which leads to questionable results.^25^

AMR genes and mutational resistance may be detected using DNA microarray technology, an alternative excellent detection approach. Microarray technology can sequentially detect a high number of different genes in a short period.^26^ DNA microarrays can be an efficient, quick, precise, robust, selective, and versatile tool for screening, diagnosing, and evaluating antimicrobial-resistant microorganisms.^27^ Initially, DNA microarrays were made with glass slides and spotted with various particular DNA probes based on reference genes found in a defined strain for which the whole-genome sequence was accessible. Comparative genomic hybridizations follow the examination of the hybridization data. Despite these factors, glass slides and fluorescent dyes enhanced the time and cost required for the procedure. It limits its applications.^8^

Considering the latest innovations in sequencing technology, whole-genome sequencing (WGS) is poised to become a critical weapon in AMR management. It has emerged as a severe concern in healthcare today.^28^ It makes it possible to identify resistance mechanisms in various bacteria in a short period. Its most significant promise, which has yet to be realized, is a vital tool for directing day-to-day infection control in hospitals and communities.^29^ This is mainly because the present technologies for automating WGS analysis lack several characteristics necessary for clinical application.

Metagenomics has shown to be a game-changing development in the field of molecular taxonomy and classification. By identifying complex microbial communities and their functional components implicated in AMR in bacteria, metagenomics has helped reveal a significant link between AMR and the microbiome.^30^ Metagenomics emerged as a standard typing approach to address the limitations of traditional culture methods in detecting uncultivable or culture-resistant bacteria. Sequence-driven and function-driven methods are used to study metagenomics data, respectively. Metagenomics is an expensive and labor-intensive process that requires exceptional abilities in wet-lab procedures, rigorous training to operate highly complex instruments, and competence in analyzing billions of sequence reads in high-throughput data processing.^31^

Sensitive molecular diagnostics such as PCR, DNA microarray, whole-genome sequencing, and metagenomics enable doctors to detect a wide variety of AMR swiftly and correctly. Although they have influenced patient care and antibiotic prescription, their impact has been limited, mainly because of concerns about bacterial coinfection and the development of resistance. Undoubtedly, the extensive and uncontrolled use of broad-spectrum and nontargeted antibiotics is a significant contributing factor to this epidemic.^32^ An example of this is the clinical indistinguishability of bacterial respiratory and fungal infections in the same patient. The speed of the detection and start of therapy also plays an essential role in disease conditions like sepsis and pharyngitis. Traditionally, antibiotic susceptibility testing requires a laboratory processing time of 48 h or more, which is sometimes impossible. As a result, doctors are forced to treat on an empirical basis, frequently using wide-spectrum antibiotics while awaiting the results of culture tests.

Further, blood cultures result in false negatives in 2% to 40% of all cases, or more, because of antecedent antibacterial therapy, fastidious organisms unable to grow on routine solid culture media (e.g., Campylobacter and *Helicobacter* species) or slow-growing anaerobes. These methods are expensive and cumbersome, but they also include a diagnostic ambiguity during which therapy is chosen based on speculation and could be suboptimal. Patients’ health and well-being are seriously impacted, and these delays lead to the establishment of AMR. Due to this, the clarity of diagnosis is always questionable.^33^

**Figure 2:**
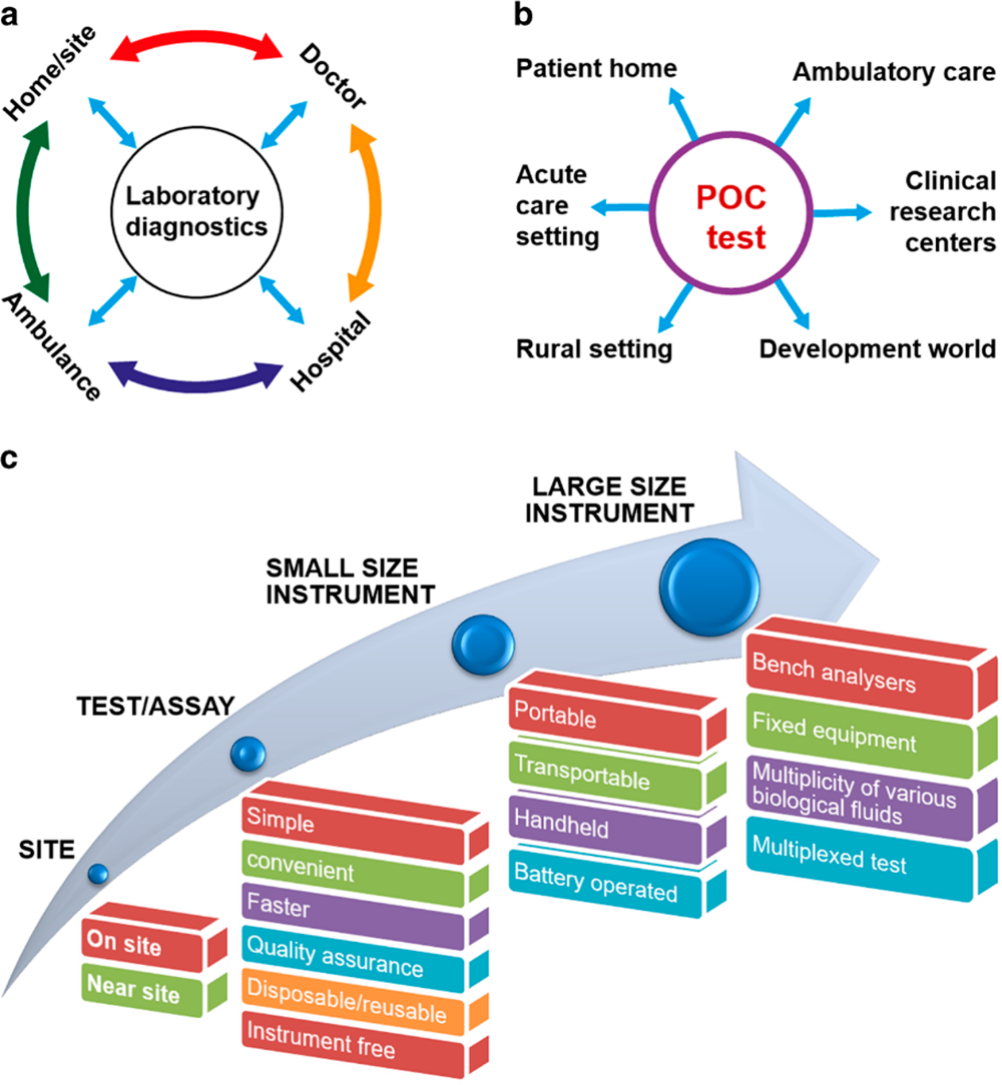
Schematic illustration of AMR diagnostic landscape. (a) Description of the routine laboratory diagnostics, (b and c) sites for POC tests and a hierarchical feature for an ideal POC test/device. Adapted under the terms and conditions of the CC-BY license from Dave, V. P.; Ngo, T. A.; Pernestig, A. K.; Tilevik, D.; Kant, K.; Nguyen, T.; Wolff, A.; Bang, D. D. *Lab Invest.***2019**, *99*, 452–469 (ref (34)).

The spread of AMR can be delayed by shortening the time it takes to diagnose the disease. Rapid diagnostics have the potential to improve both the treatment and care of infected individuals. In routine laboratory diagnostics, testing is often carried out in a laboratory environment, away from the specific patient. However, on the other hand, patients can benefit from point-of-care technology in a variety of situations ranging from basic physical testing to bedside diagnostic tests with low resource rural settings, as shown in Figure 2.^34^

AMR is tough to control due to a lack of sensitive and precise diagnostic tests. Choosing the best appropriate drug, on the other hand, might be difficult in the absence of a specific and sensitive diagnosis method. Rapid and accurate diagnostic testing can save patients’ lives by reducing the time it takes to administer appropriate antibiotics, reducing the use of antibiotics that are not necessary, and informing decisions about antibiotics, such as which drugs to use, what doses to prescribe, and how long to prescribe them.^8^

Rapid identification and characterization of harmful micro-organisms are essential to minimize disease transmission and inform clinicians of patient treatment regimens. Effective treatment methods can be guided by quick diagnostic tests that identify antimicrobial-resistant bacteria, establish the mechanism of developing AMR, and differentiate viral from bacterial infections. Rapid diagnostic tests may also help with epidemiological surveillance by monitoring the development of resistant infectious pathogens and their spread.

The demand for nanotechnology-based POC is expanding in response to the growing interest of patients in self-management and self-monitoring. The invention of upgraded biosensors that incorporate cutting-edge nanomaterials and nanotechnology represent potential diagnostics methods of the future.^35,36^ The use of nanomaterials in diagnostics can increase the sensitivity and precision of pathogen detection. It will further reduce unnecessary hospitalizations and antibiotic prescriptions. It allows clinicians to take an objective, straightforward approach to their clinical assessments of the patient’s symptoms and indications. Primary care prescribers may employ nanomaterials-based diagnosis to inform disease management, mainly if these tests can be completed during a patient visit. Adopting nanomaterials-based diagnostics may enhance antibiotic usage and minimize patient demand for antibiotic prescriptions.^37^ POC diagnostics have played a significant role in the evolution of the healthcare industry during the last few decades.

Nanotechnology-based technologies provide many critical practical benefits compared to conventional techniques, including improved surface reactivity, quantum confinement effects, higher electrical conductivity, and enhanced magnetic characteristics.^38^ Thus, we may conclude that nanodiagnostics involves developing systems that utilize nanostructures to personalize diagnoses. On the other hand, the combination of such devices with nanomaterials paves the way for developing highly sensitive and selective biosensors for the next generation of POC diagnostics.

## Innovation in the Field of Diagnostics by the Engineering of Materials

A common antagonist of antiviral, antibacterial, and anticancer medicines is the development of drug resistance. Large numbers of individuals suffer from bacterial infections, resulting in substantial consequences for their quality of life and healthcare costs. The proliferation of microorganisms capable of AMR is a global issue, owing to a scarcity of antibiotics accessible to treat multidrug-resistant bacterial infections in people and animals.^21^ The conventional techniques for pathogen detection, including antibody-based assays and those that amplify nucleic acids for detection, have essentially hit their high sensitivity and specificity.

Researchers are motivated to develop economically feasible, efficient, accurate and cost-effective options utilizing advanced technology to alleviate the restrictions associated with accessibility. This vacuum may be filled by designing and developing materials that strike the right balance between excellent quality and affordability. The interaction between nanotechnology and microorganisms offers a new quest to combat human diseases.

Nanotechnology has the potential to revolutionize both the diagnosis and treatment of AMR. These technologies, which include systems with a diameter of roughly 1000th of a hair’s thickness, significantly affect world morbidity and mortality causes.^39^ Nanoscale materials, defined as those having at least one dimension on the order of 1–100 nm. Due to its size-dependent optical, electrical, physical, and chemical characteristics, it has attracted considerable interest for application in improving diagnostic systems. Numerous nanomaterials have been used for detection, therapy, or theranostic applications due to their distinctive thermal, magnetic, optical, or redox potentials.^40^ Hence, the use of nanoparticles (NPs) in biodetection is vast. NPs platforms are providing new insights into pathogen detection and management of therapies.

However, there are still evident shortcomings in selectivity, durability, and other elements of sensitive material development at present; as a result, the development of innovative high-performance detectors will have a significant realistic meaning and practicability. Given the engineering and nanomaterials involved in AMR detection, further work on sensor development will necessitate a thorough knowledge of functional nanomaterials. Numerous nanomaterials, including noble metals nanoparticles, quantum dots (QDs), lanthanide nanoparticles, silica nanoparticles, carbon nanomaterials, dendrimers, and magnetic nanoparticles, have been utilized recently to produce nanotechnology-based fast diagnostic tests.^41^ The characteristics of nanomaterials employed in diverse biosensing applications play a role in their selection. The unique physiochemical features of nanomaterials can identify novel antimicrobial targets.^42^

### Consideration and Overview of Materials for Detection of AMR: A Concurrent Engineering Perspective

Even though nanotechnology has been promoted as the panacea for many scientific problems, it has only just begun to deliver on its promise after years of scientific research. Many cutting-edge technologies have emerged in this rapidly growing sector and hold the potential to enhance diagnostic capabilities for chemical and biological agents and assist in the identification of disease biomarkers. The majority of nanomaterials are chosen depending on the following properties (i) increase the sensitivity and specificity of tests and limits of detection (LOD), (ii) to increase the number of samples processed, and (iii) to decrease the complexity and expense of the assay. The nanomaterials-based emerging technology with an improved biosensors detection limit will provide a platform for next-generation biosensors.^43^ This section provides an overview of several engineered nanomaterials that can be used to detect AMR.

#### Noble Metallic Nanoparticles

**Figure 3:**
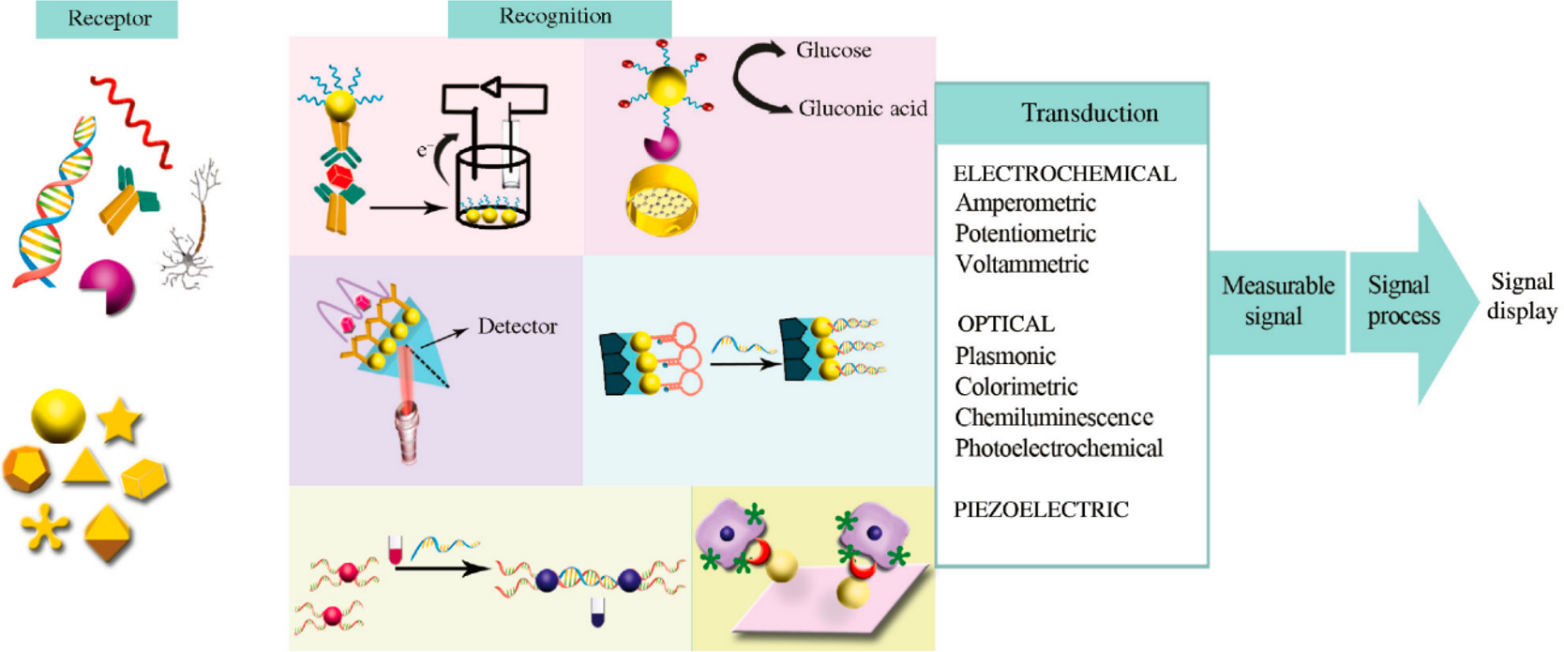
Schematic presentation of the role of noble MNPs in numerous biosensors. Adapted under the terms and conditions of the CC-BY license from Malekzad, H.; Sahandi Zangabad, P.; Mirshekari, H.; Karimi, M.; Hamblin, M. R. Noble Metal Nanoparticles in Biosensors: Recent Studies and Applications. *Nanotechnol Rev.***2017**, *6* (3), 301–329 (ref (44)).

Noble metal NPs are particularly well suited to biomedical applications like optical contrast agents, multimodal sensors that combine optical and scattering imaging, and photothermal treatment, as shown in Figure 3.

For several decades, scientists have been enthralled with noble metallic NPs such as gold nanoparticles (AuNPs), silver nanoparticles, and platinum nanoparticle (PtNPs), partially due to colorful colloidal solutions.^45^ While various noble metal nanoparticles are used for detection, the most thoroughly investigated and most commonly utilized are AuNPs and AgNPs. They have outstanding optical, physical, chemical, and biological characteristics and have been studied extensively. The simplicity of operation, the cost-effectiveness of manufacturing, and the sensitivity of the developed materials make them suitable candidates for developing detection devices.^46^ Surface area and particle size are critical factors in improving the sensitivity and selectivity of the detection technique. AuNPs and AgNPs have an extensive surface area and small particle size crucial for increasing detection technique selectivity and sensitivity. The characteristics of these NPs can be altered if sensors based on AuNPs and AgNPs contact the target molecule. Accordingly, visual signals of electrochemical or optical signals may be generated, linked with the analyte concentration, and used to investigate the response further. Consequently, the reaction may be utilized to determine the presence and amount of specific substances.^47^ Synthesis and detection of diseases by using AuNPs^48,49^ and AgNPs have been well established by different researchers.^50,51^ There is considerable demand for highly refined gold nanoparticles in bioassays because of their capability to regulate particle sizes in the forms of spheres carefully, cubes, rods, and wires.^52−54^

AuNPs can support numerous detection platforms, i.e., a target analyte may be detected using more than one detection approach, such as spectroscopic, colorimetric, fluorimetric, and electrochemical methods.^55^ Gold nanoparticles may be functionalized with antibodies or another ligand of interest to target a pathogen of interest. The target DNA hybridization is utilized with complementary probes in most selectivity biosensors, significantly reducing the detection time. A very sensitive fluorescent nanobiosensor was developed by Elahi et al. for detecting *Shigella* species.^56^ DNA probes and AuNPs were designed to fulfill this objective. Then, as a signal reporter, two DNA probes were fixed on the surface of AuNPs. The fluorescent DNA probe was applied to the surface of AuNPs, and the fluorescence intensity was measured using fluorescence spectrophotometry. The technique detected bacteria at low quantities (10^2^ CFU mL^–1^).^56^

Silver nanoparticles offer various beneficial optical characteristics that have paved the way for novel sensing and imaging applications. The advantage of the detection system is that it provides a broad range of detection modes, including colorimetric, scattering, SERS, and MEF methods, all at very low detection limits.^57^ Chemical stability, high conductivity, and outstanding optical properties are some of the advantages of AgNPs.^58^

#### Quantum Dots

Quantum dots (QDs) are inorganic semiconductor crystals with a nanometer-scale system composed of elements from groups II–VI or III–V. Basically, in the periodic table, II–VI (e.g., Cd, Zn, Se, and Te) or III–V (e.g., In, P, and As).^59^ CdSe, CdTe, HgTe, PbS, PbSe, PbTe, InAs, InP, and GaA are examples of this.^60,61^ It has a diameter that generally ranges between 2 and 6 nm. In this size range, quantum confinement allows for the formation of highly discretized band structures, resulting in emission wavelength shifts proportional to the nanocrystal size.^36^ Their novel new characteristics, which include improved brightness and optical properties,^62,63^ size-tunable emissions from visible to NIR,^64^ high quantum yield,^65^ long fluorescent lifetimes,^66^ narrow emission spectra,^67^ and high resistance to photobleaching,^68^ are particularly appealing. Because of their distinct characteristics, they have found widespread applications in biosensing applications.

Moreover, they have opened up new opportunities for ultrasensitive analytical and imaging techniques. QDs have gained popularity as reporter labels in biosensing applications because of their unique and highly desired luminous characteristics. QD sensors that work by manipulating fluorescence resonance energy transfer (FRET) are exciting because they may employ a variety of response mechanisms, allowing for more design flexibility. Additionally, they can be used as ratiometric or “color-changing” probes.^69^ A positively charged QD-based FRET probe for detecting micrococcal nuclease was developed by Qiu et al. by taking advantage of QD-FRET probes sensitivity.^70^ Under optimal circumstances, the ratio is linearly related to the concentration of micrococcal nuclease (MNase) throughout the range of 8 × 10^–3^ to 9.0 × 10^–2^ unit mL^–1^, with an LOD of 1.6 × 10^–3^ unit mL^–1^. A novel detection strategy is straightforward to use, allowing it to be applied in DNA-related bioassays that use the FRET using positively charged QDs-based reagents.^70^ Later on, Qiu and Hildebrandt (2015) have shown a QD-FRET test that can measure three different miRNA from clinical samples down to 0.3 pM.^71^ In the field of flow cytometry, QDs are expected to have the most considerable influence. It gives the ability to conduct highly complex tests and to improve the resolution of faintly stained markers. Flow cytometry investigations are quick, affordable, and multianalyte-capable. Multiplexed flow cytometry and simultaneous detection of several distinct QDs are possible using QDs with broad excitation and narrow emission bands. Flow cytometry-based on quantum dots is an efficient method for pathogen identification. QDs were utilized in one investigation to detect the respiratory syncytial virus (RSV) and the relative concentrations of RSV surface proteins in different viral strains.^72^

#### Lanthanide Nanoparticles

**Figure 4:**
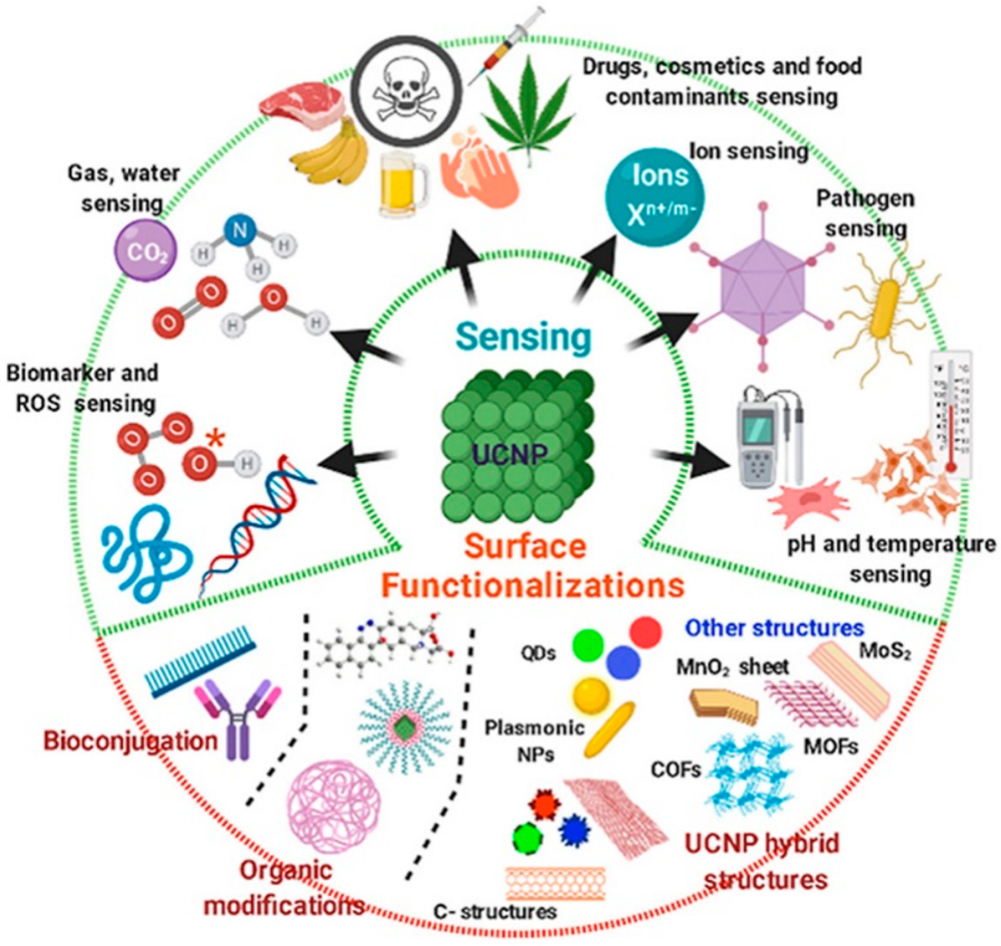
Application of lanthanide nanoparticles in sensing. Reproduced from Kumar, B.; Malhotra, K.; Fuku, R.; Van Houten, J.; Qu, G. Y.; Piunno, P. A. E.; Krull, U. J. *TrAC–Trends Anal. Chem.***2021**, *139*, 116256 (ref (76)). Copyright 2021, with permission from Elsevier.

Lanthanides with distinctive photophysical characteristics, such as europium, terbium, and ytterbium, make them useful molecular probes of biological systems.^73^ Lanthanide luminescence is mainly characterized by lanthanide ions that are incredibly long-lived (microseconds-to-milliseconds) in luminescence than standard nanosecond-level dyes. Lanthanide-doped nanoparticles exhibit extraordinary luminous characteristics, including a broad absorption shift, a narrow emission bandwidth, resistance to optical blinking and photobleaching, and the capacity to convert long-wavelength stimulation to short-wavelength emission.^74^ Lanthanide complexes are often employed as biological fluorescent tags, and commercial signal detecting equipment is widely available in laboratories and hospitals. The emission of NPs is strong, and the detection sensitivity is very high. Due to the extended luminescence lifespan of NPs and the time-resolved (TR) imaging method, compassionate target identification is possible without interruption from the background noise.^75^ Because of their increased sensitivity, lanthanide compounds are becoming increasingly attractive alternatives to traditional fluorescent dyes in diagnostic applications. Due to the well-established benefits of lanthanide-doped nanoparticles, it has been widely employed for detecting a wide variety of analytes in recent years, as shown in Figure 4.^76^ Toro-González et al. reported lanthanide phosphate nanoparticles (NPs) radiolabeled with ^156^Eu with low toxicity, resistance to radiation, and unusual luminescent and magnetic characteristics make this compound ideal for biological applications.^77^

#### Silica Nanoparticles

It has been demonstrated that silica-based nanoparticles may be produced and doped with organic and inorganic dye molecules and fabricated to include magnetic cores encapsulated in silica covering.^78^ Due to the inherent surface chemistry of silica, it is possible to functionalize silica nanoparticles with various functional groups such as amino, carboxyl, thiol, and methacrylate.^79,80^ Several methods, such as layer-by-layer assembly, physical adsorption, and silane coupling agents, are widely employed.^81,82^

Molecularly engineered mesoporous silica nanoparticles (MSNs) are powerful nanoparticles-based platforms for detecting and monitoring bacterial infections. MSNs allow the use of multimodal imaging modalities to be combined into a single MSN system. Similar tactics have already been used with other types of nanoparticles for precise, selective, and rapid bacterial detection and labeling by altering the surfaces of the nanoparticles.^83^ An easily synthesized, porous silicon-based biosensor was developed for fast bacterial detection. Silicone (0.01-ohm cm, p-type) has been electrochemically anodized to form the spongelike porous silicon layer in an electrochemical Teflon cell containing ethanoic hydrofluoric acid. The *Escherichia coli* (*E. coli*) and enzyme reaction with the dioxetane substrate resulted in light production at 530 nm. The porous silicon biosensor chip containing *E. coli* emitted considerably more light than the control and planar silicon biosensor chips containing *E. coli* (*P* < 0.01). The reported sensitivity of porous silicon biosensor was 10^1^–10^2^ colony forming units (CFU) of *E. coli*. The newly designed biosensor can help to identify *E. coli* in food and environmental tests.^84^

#### Carbon Nanomaterials

Carbon-based materials have a long history, dating back to the 1950s when the first research projects on Radushkevich and Lukyanovich were completed. Semiconductors, based on graphite, were used in the Space Race throughout the 1960s. In the 1990s, researchers at the Massachusetts Institute of Technology created a new type of material that could be utilized to produce solar cells.^85^ Novel carbon-based materials have widely been preferred for biosensor development due to outstanding physicochemical characteristics, such as high mechanical strength, high conductivity, appealing optical qualities, chemical flexibility etc.^86,87^ Consequently, it has found use in the areas of electronics, materials science, and chemistry. The introduction of carbon nanoparticles such as carbon nanomaterials (CNTs) and graphene has been used extensively to create novel electrical and biosensor sensors.^88^

Nanostructured materials, particularly carbon nanotube (CNT)-based sensing cues for analytical detection applications, are of particular interest. CNTs are classified into single-walled carbon nanotubes (SWCNTs) and multiwalled carbon nanotubes (MWCNTs). MWCNTs are numerous concentric tubes of graphene surrounding one another, while SWCNTs are seamless one-dimensional cylindrical tubes made from a single graphene sheet. The design of carbon nanotubes has extraordinary electrical, mechanical, biological, and thermal properties, making them ideal for critical real-time applications with exceptional performance.^89^ CNTs may be functionalized covalently or noncovalently with biorecognition components. The most frequent functionalization method is to expose oxides on the surface of the CNTs by treating them with acids. CNTs are often incorporated into field-effect transistors (FETs) and utilized as electrochemical sensors for DNA, proteins, cells, and other pathogen biomarkers.^36^ Because of the increased surface area, CNTs may improve the electrochemical response observed when a biorecognition element and target react and the superior electrocatalytic activity provided by exposed graphite edge planes.^90^ Munawar et al. developed a novel nanohybrid material in which 3D imprinted nanostructures were embedded.^91^ In this study, this material was used to construct an electrochemical sensor used to monitor an experimental veterinary medication, chloramphenicol. The excellent transmission and conductivity of electrons in the developed material resulted in a sensitive response. It has been shown that altering the polymer composition, the amount of cross-linking, and the thickness of the sensor layer significantly impact the number of binding sites available for drug molecule identification. This study opens the door for the development of variations of three-dimensional imprinted materials for the detection of additional biomolecules and antibiotics.^91^

To detect the presence of *Bacillus cereus* DNA sequences, Zuo et al. designed a label-free DNA biosensor based on magnetite/multiwalled carbon nanotubes/chitosan (Fe_3_O_4_/MWCNTs-COOH/CS) nanomaterial.^92^ Cyclic voltammetry (CV), differential pulse voltammetry (DPV), and electrochemical impedance spectroscopy (EIS) were used to carry out the electrode surface and hybridization procedure. Under ideal situations, the biosensor demonstrated an excellent linear relationship between the peak currents difference and the logarithm of the target DNA concentration. It ranges from 2.0 × 10^–13^ to 2.0 × 10^–6^ M with a detection limit of 2.0 × 10^–15^ M.^92^

Graphene is one of the most promising and popular strategies for bottom-up nanotechnology techniques. It has grown to be one of the most active research areas in recent years. Graphene is the basic building block for various carbon allotropes such as graphite, charcoals, carbon nanotubes, Buckminsterfullerene and other buckyballs, and so on.^93^ Graphene is gaining popularity in the physical, chemical, and biological sectors as a new nanomaterial with numerous unique properties. It includes incomparable thermal conductivity (5000 W m^–1^ K^–1^), exceptional electrical conductivity (1738 Siemens per m), high surface-to-volume ratio (2630 m^2^ g^–1^), remarkable mechanical strength (about 1100 GPa), and biocompatibility.^94^ Reduced graphene oxide (rGO), graphene (G), and graphene oxide (GO) have incredibly high fluorescence quenching efficiency. Thus, graphene-based nanomaterials may also be utilized as a quencher to make fluorescent transducer-based biosensors. Graphene affects the detection limit of targeted molecules during sensor design, and bioreceptors may also influence the sensitivity and selectivity of biosensors and the orientation of the G, GO, or rGO sheet during sensor design.^95,96^ There are differences in the detecting capability of biosensors based on functional groups, graphene oxidation state, number of layers, and derivatives utilized.^97^ Recently, graphene and functionalized graphene have been used effectively in various electrocatalysis and electrochemical biosensing applications, demonstrating significant promise. Akbari et al. fabricated three distinct models to characterize the *I*–*V* relationship of a graphene-based sensor for *E. coli* bacteria.^98^ These models included an artificial neural network (ANN), support vector regression (SVR), and an analytical approach. When exposed to *E. coli* bacteria at concentrations ranging from 0 to 10^5^ CFU/mL, the graphene device’s conductivity increases dramatically by orders of magnitude. The simplicity, rapid reaction time, and high sensitivity of this nanoelectronic biosensor make it a perfect device for sensitive detection of antibacterial drugs as well as an excellent high-throughput platform for the detection of any harmful pathogens.^98^

#### Dendrimers

**Figure 5:**
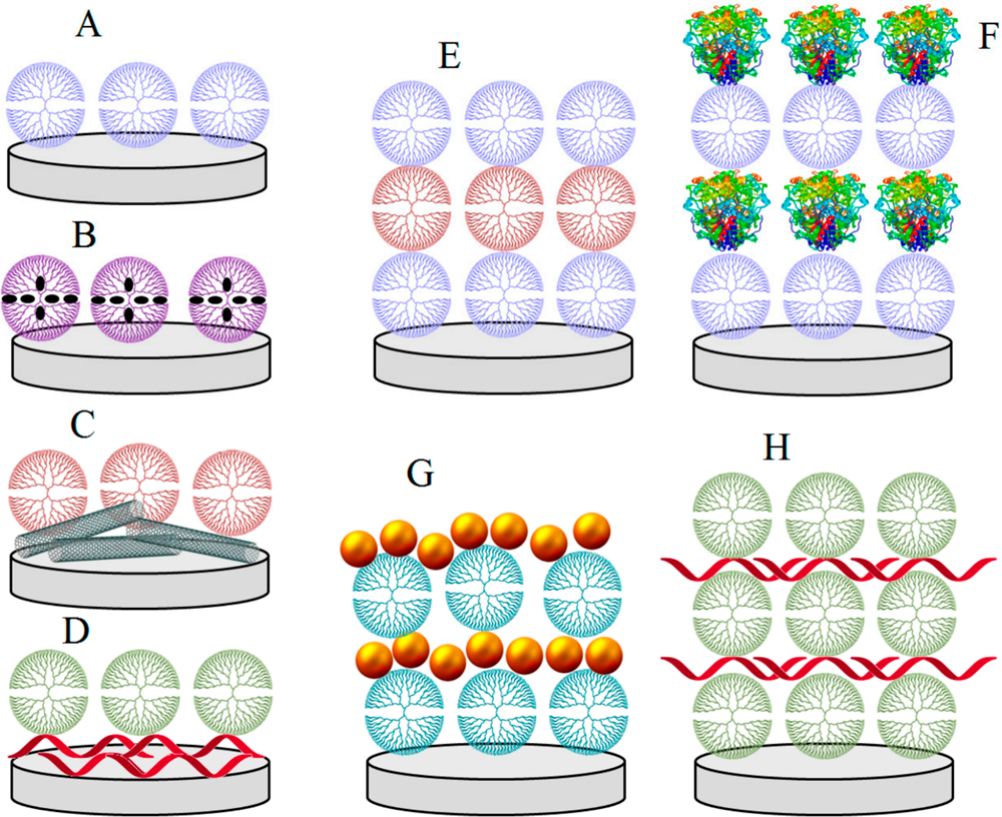
Assemblies of dendrimers on electrode surfaces: (A) molecularly organized dendrimer monolayer, (B) monolayer of metal nanoparticle-decorated dendrimers, (C) dendrimer layered on the nanomaterial-modified surface, (D) dendrimer layered on the polymer-coated surface and layer-by-layer assemblies of (E) dendrimer/dendrimer, (F) dendrimer/protein, (G) dendrimer/nanoparticles, and (H) dendrimer/polymer bilayers. Adapted under the terms and conditions of the CC-BY license from Sánchez, A.; Villalonga, A.;Martínez-García, G.; Parrado, C.; Villalonga, R. Dendrimers as Soft Nanomaterials for Electrochemical Immunosensors. *Nanomaterials***2019**, *9* (12), 1745 (ref (103)).

Dendrimers are nanoscale polymeric structures with a high density of surface functional groups that are monodispersed, three-dimensional, and hyperbranched. These molecules have a defined molecular weight, shape, and size, making them ideal molecules for a wide range of applications in various fields.^99^ Dendrimeric platforms have been effectively utilized to detect proteins, DNA, pathogens, chemicals, and other molecules using different sensor methods, such as electrochemical sensors,^100^ fluorescence,^101^ gravimetric,^102^ etc. An ideal conductive surface is required for electrochemical detection for it to function correctly. Even though dendrimers are not well-known for being excellent conductors, metallic compounds or colloids may be readily linked to their numerous functional groups to increase their conductivity. Several assembly methods may be used to build dendrimer-based 3D layer arrangements on an electrode surface, including molecularly structured monolayers and a wide range of hybrid layers when polymers and nanomaterials are mixed. These fractal-like macromolecules may also be used to construct ordered layer-by-layer structures with other dendrimers, proteins, polymers, and “hard” nanomaterials. Figure 5 depicts a few examples of potential configurations for this situation.^103^

Lu et al. reported developing a new electrochemical immunosensor for *E. coli* detection in urban sludge based on dendrimer-encapsulated Au and enhanced gold nanoparticle labeling.^104^ Using an electropolymerization process on GCE, they discovered *p*-aminobenzoic acid (p-ABA) produced numerous carboxyl groups. Gold nanoparticles (AuNPs) were subsequently reduced in the dendrimer’s interior to produce Au(III) ions. The coordination of Au(III) ions in the dendrimer’s interior was followed by reduction, resulting in gold nanoparticles (AuNPs). The resultant electrode (GCE/p-ABA/PAMAM (AuNPs)) included many amino groups, enabling extremely dense immobilization of *E. coli* and improved electrochemical performances.^104^

#### Magnetic Nanoparticles

Magnetic nanoparticles (MNPs) that combine the characteristics of noble metal nanoparticles (NPs) with magnetism.^105^ MNPs differ in their chemical, mechanical, and magnetic characteristics, especially in conventional micro- and macromaterials, because of the exclusive size effect.^106^ Iron (Fe) and other ferromagnetic materials have a magnetization value (Ms) that may be determined via vibrational sample magnetometry (VSM).^107^ For biomedical applications, however, the element iron in the form of either maghemite (Fe_2_O_3_, γ-Fe_2_O_3_) or magnetite (Fe_3_O_4_) has been more often used for detection.^108^ Bhattacharya et al. demonstrated a fast, sensitive, specific, and effective technique for detecting harmful bacteria at ultralow concentrations by utilizing antibody-labeled multifunctional Au–Fe_3_O_4_ nanocomposites in conjunction with a fluorescent probe.^109^ When bacteria were exposed to probes, the fluorescence and optical pictures of the bacteria revealed that the pathogen bacteria were first identified and then eliminated from the *Staphylococcus aureus* (*S. aureus*) solution within 30 min of contact. *S. aureus* may be immunomagnetically collected, identified, and eliminated within 30 min at a concentration of 10^2^–10^7^ CFU mL^–1^. Antibody-targeted nanoprobes can be regarded as a new toolbox for the rapid, specific, and sensitive detection of particular organisms like *S. aureus*.^109^

#### Metal–Organic Frameworks (MOFs)

Metal–organic frameworks (MOFs) are porous coordination materials of multidentate organic ligands and metal ions or metal clusters. They are used in a wide range of applications.^110^ It possesses exceptionally effective luminous sensors (both chemo- and bio-) for various analytes, including cations, anions, emerging contaminants, gases, and biomolecules.^111^ In comparison to other fluorescent nanomaterials such as quantum dots and metal nanoparticles, MOFs have a higher surface area, improved photostability, increased fluorescence yield, adjustable and accessible pores, and readily available functional groups.^112,113^ Gupta et al. published a paper describing the optical detection of *E. coli* using a water-dispersible terbium MOF (Tb-BTC).^114^ The biosensor detects analytes with concentrations ranging from 1.3 × 10^2^ to 1.3 × 10^8^ CFU/mL, with a 3 CFU/mL detection limit.^114^ Duan et al. synthesized copper-based MOF nanoparticles (Cu-MOF NPs) and functionalized them with aptamers to create a colorimetric technique for detecting *E. coli*.^115^ The immobilization of aptamer 1 onto a microplate to serve as capture probes in a typical experimental approach. To generate the signal probes, Cu-MOF NPs were produced and functionalized with streptavidin and biotinylated aptamer 2. Both capture and signal probes’ aptamers bind with *E. coli* and form a sandwich-type complex with the aptamers. Cu-MOF NPs can catalyze the colorless peroxidase substrate, resulting in the production of a colorimetric output signal. The colorimetric aptasensor showed a rapid and sensitive quantification of *E. coli* in the concentration range of 16–1.6 × 10^6^ CFU/mL with a limit of quantitation (LOQ) of 16 CFU/mL and limit of detection (LOD) of 2 CFU/mL.^115^

## Detection Modalities: A Trend toward POC Based AMR Detection

The development of resistance eventually reduces the efficacies of all antibiotics against various bacteria. Such infectious diseases have the potential to cause significant mortality and morbidity. It accentuates the need of detecting and evaluate pathogenic microorganisms as early and efficiently as possible. Timely screening of AMR would allow for the introduction of early intervention options, which would either slow down the course of the disease or prevent the start of substantial mortality and morbidity from occurring altogether. Various approaches to developing diagnostic platforms include electrical, mechanical, nuclear magnetic resonance (NMR), electrochemical, and optical-sensing technologies. However, here we cover the most recent advancements in biosensors for pathogen detection, focusing on optical and electrochemical-based biosensors, and discuss the technologies and strategies that enable such optical and electrochemical-based biosensors to fulfill these detection functions. Advances include implementing microfluidic samples, portable data processing and multifunctional materials to increase sensitivity, specificities and simplicity of operation. This paper presents recent examples of optical and electrochemical biosensors, along with their advantages and limitations. The electrochemical and optical-based detection for AMR is summarized in Table 1.

**Table 1:** Summary of Recent Optical and Electrochemical Based POCT for Pathogen Detection

pathogen	LOD	signal transduction modality	detection time	ref
*S. aureus*	80 CFU/mL	fluorescence	45 min	(116)
*S. aureus*	3.1 CFU/mL	optical fiber biosensors	40 min	(117)
*E. coli*	10^2^ cfu mL^–1^	lateral flow immunoassays		(118)
*S. aureus*	3.1 CFU/mL	optical fiber biosensors	30 min	(117)
*E. faecalis*	down to ∼100 bacteria/mL	plasmonic sensor		(119)
*V. parahemolyticus*	10^2^–10^7^cfu/mL	colorimetric		(120)
*E. coli*	5 mM	colorimetric	8 h	(121)
*S. aureus*	10 CFU/mL	FRET	30 min	(122)
*C. trachomatis* and *N. gonorrheae*	300 CFU/mL for *C. trachomatis* and 1500 CFU/mL for *N. gonorrheae*	nanoplasmonic biosensor	<1 h	(123)
MRSA	2 × 10^0^ CFU per 100 g	PCR-LFI	3 min	(124)
*Salmonella choleraesuis*	5 × 10^5^ CFU per mL	LFIA		(125)
ciprofloxacin	0.028 nM	CV		(126)
MRSA	5 CFU mL^–1^	CV		(20)
*E. coli*	2 × 10^3^ CFU/mL	CV	30 min	(127)
*V. parahemolyticus*	5.3 × 10^–12^ M	CV	10 min	(128)
*E. coli* and *V. cholera*	39 CFU/mL and 32 CFU/mL	CV		(129)
*Vibrio parahemolyticus*	2.16 × 10^–6^ μM	electrochemical biosensor		(130)
*Salmonella typhimurium*	3 CFU mL^–1^	impedimetric biosensor	45 min	(131)
aflatoxin B1	0.4 nM	CV	10 min	(132)
*E. coli*	102 to 103 CFU/mL	EIS	30 min	(133)

### Optical Detection

The development of biosensors for POC is a continuing trend in pathogen detection. Optical biosensors have demonstrated commendable efficacy in detecting biological systems, paving the way for substantial advancements in clinical diagnostics in recent times.^134^ Optical biosensors are inexpensive diagnostic instruments that allow direct, fast, and label-free detection of bacterial infections compared to traditional methods. Because of their ease of operation, high sensitivity, and quick detection, healthcare has widely accepted optical sensors to detect AMR.^135,136^ In recent years, there has been a lot of interest and excitement about the increasing availability of diverse nanocarbons with unique and finely tuned optical properties as well as their excellent performances in bioimaging both in vivo and in vitro. This has prompted many researchers to consider their potential application in bacterial recognition and quantification.^137,138^ CNPs or carbon dots (CDs) are new nanomaterials that show intrinsic optical fluorescence and are becoming more popular. It is possible to detect bacteria using nanocarbons and their derivatives based on fluorescence, imaging, and color change. This is accomplished by the interaction between fluorophores and bacteria, facilitated by the various binding domains found in nanocarbons. The unique optical characteristics of CNPs enable them to adjust the location and strength of their emission peak. This may be accomplished by either stimulating the CNPs at different wavelengths or by introducing external stimuli such as pH, temperature, or the presence of the particular analytes in the solution to the CNPs.^139−142^ The refractive index-based optical sensors encompass various technologies such as colorimetric, surface-enhanced Raman scattering, immunochromatographic assays (ICAs), and plasmon based technology.^143^ The sensitivity of optical sensing platforms has been enhanced, making them more appropriate for detecting small quantities in clinical samples.

#### Colorimetric Detection

**Figure 6:**
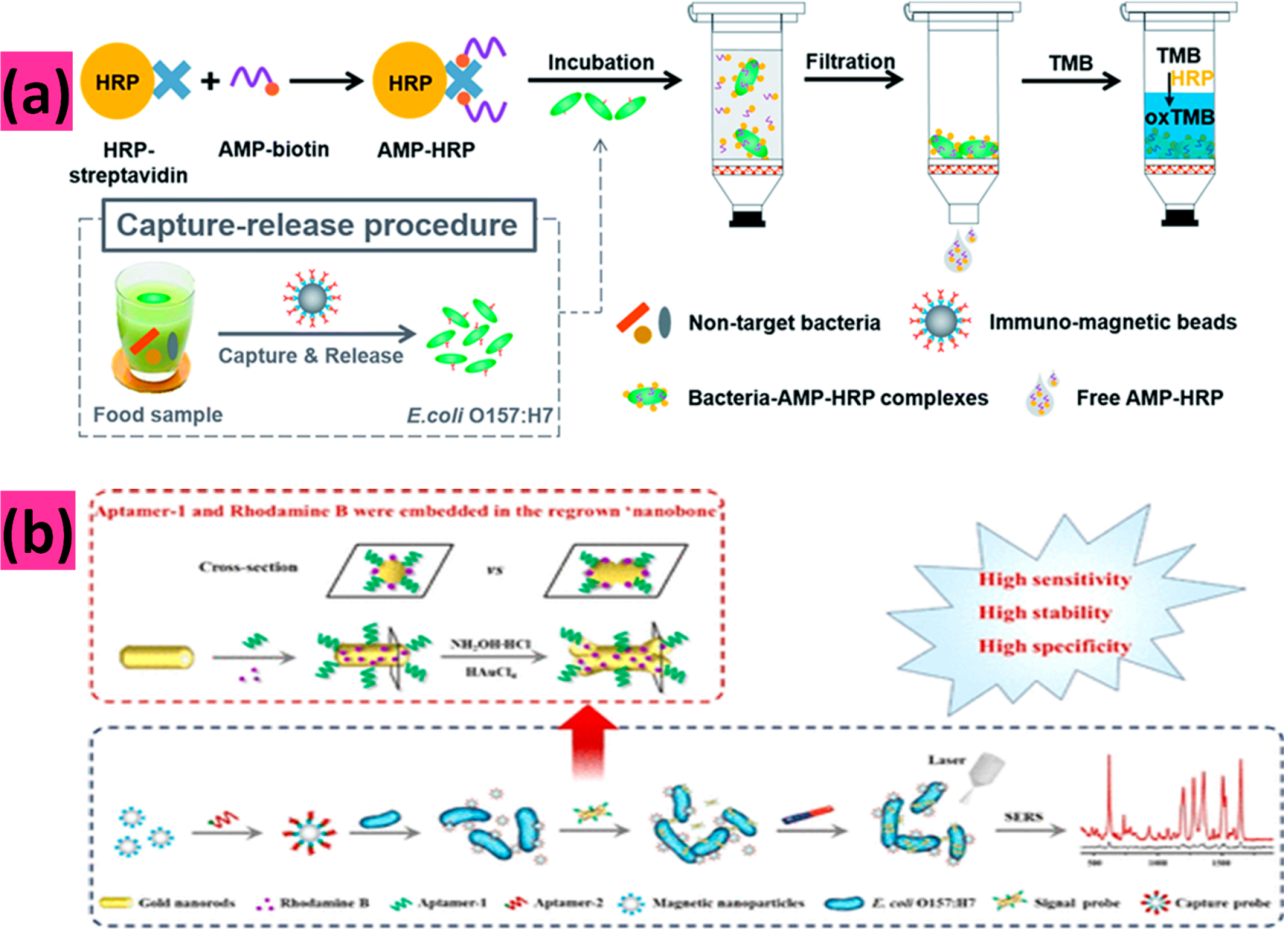
Colorimetric detection of *E. coli* bacteria. (a) The antimicrobial peptide-based colorimetric bioassay for detecting *E. coli*. Reproduced from Qiao, Z.; Lei, C.; Fu, Y.; Li, Y. An Antimicrobial Peptide-Based Colorimetric Bioassay for Rapid and Sensitive Detection of *E. coli* O157:H7. *RSC Adv.***2017**, *7*, 15769 (ref (144)). Copyright 2017, with permission of The Royal Society of Chemistry. (b) SERS based detection of *E. coli.* Zhou, S.; Lu, C.; Li, Y.; Xue, L.; Zhao, C.; Tian, G.; Bao, Y.; Tang, L.; Lin, J.; Zheng, J. *ACS Sensors***2020**, *5* (2), 588–596 (ref (147)). Copyright 2020 American Chemical Society.

Colorimetric responses of bacterial cell identification can be detected with the naked eye or by using simple spectroscopic techniques, depending on the situation. Qiao et al. developed an antimicrobial peptide (AMP)-based colorimetric bioassay for the fast and sensitive detection of *E. coli* O157:H7 bacteria.^144^ Instead of using antibody-HRP, AMP was coupled with horseradish peroxidase (AMP–HRP) to produce a signal reporter with greater sensitivity than previously available. Because of the abundance of AMP-binding sites on the surface of target bacteria, the suggested bioassay could detect *E. coli* O157:H7 at concentrations as low as 13 CFU mL^–1^ in pure culture with a linear range of 10^2^–10^5^ CFU mL^–1^ in 45 min without the need for pre-enrichment. As demonstrated in Figure 6a, sensitive and selective deletion of *E. coli* was performed in combination with immunomagnetic capture-release.^144^

#### Surface-Enhanced Raman Scattering (SERS)

SERS is a powerful technique that depends on interactions between a molecule and a nanostructural metal surface, causing an increase in the Raman signal.^145^ The usage of SERS has attracted scientists because of this characteristic, and it is capable of providing real-time detection and on-site sensing. There are several articles in which SERS is utilized in biomedical applications, including detecting a biomolecule, blood testing, and detecting cancers.^146^

Zhou et al. demonstrated the detection of *E. coli* O157:H7 with great sensitivity, robustness, and specificity.^147^ The preparation of multifunctional gold nanobones (NBs) (GNRApt-1+RhB) from gold nanorods (GNRs) is mediated by an aptamer (Apt-1) and the signal molecule rhodamine B (RhB) by the one-pot step method. The NBs (GNRApt-1+RhB) are used for surface-enhanced Raman scattering detection of *E. coli*. The Raman amplification was caused by a high electromagnetic field distribution at the apex of both GNRApt-1+RhB ends. The signal stability was caused by the solid embedding of Apt-1 (poly A20 + *E. coli* O157:H7 aptamers) and RhB on the GNRApt-1+RhB surface. Optimization experiments revealed that surface-enhanced Raman-scattered RhB absorption at 1350 cm^–1^ exhibited a strong linear relationship (*y* = 180.30*x* – 61.49; *R*^2^ = 0.9982) with *E. coli* O157:H7 concentrations ranging from 10 to 10 000 CFU/mL with a limit of detection of 3 CFU/mL as shown in Figure 6b.^147^ This combination demonstrated excellent identification, stability, and a substantial increase in Raman signal intensity.

#### Immunochromatography

Immunochromatography is also referred to as lateral flow immunoassay (LFIA). It is a straightforward, quick, convenient technique that allows portability. Although this technique has been in use for several decades, recent improvements in its sensitivity, reproducibility, and ability to detect multiple analytes have made LFIA an attractive option for diagnosing hospital-acquired (nosocomial) infection.^148,149^ Noble metal nanoparticles (NMNPs) have generally been utilized in LFIAs due to their capability of providing a diagnostic signal visible to the naked eye, removing the need for an external excitation source or emission sensor.^150^ Based on the principle of colloidal gold immunochromatography, Kong et al. reported the simultaneous detection of *Haemophilus influenzae* (*H. influenzae*).^151^ Transmission electron microscopy and ultraviolet–visible spectroscopy (200–700 nm) were used to confirm their findings. The test strip was constructed on a plastic backing that included a sample pad, a conjugate pad, an absorbent pad, and a nitrocellulose membrane onto which the test and control lines have adhered. The strip demonstrated specific recognition of *H. influenzae* but did not demonstrate recognition of any other common respiratory pathogens. The detection limit for the test line was as low as 1 to 10^6^ CFU per mL, and the entire procedure could be finished within 10 min. The strips could be kept at 4 °C for 6 months without compromising their sensitivity or specificity.^151^

#### Plasmonic Based Sensor

Sensors based on plasmonics are perhaps the most well-known and extensively preferred sensors. Plasmonic-based systems have recently emerged as a promising contender for developing next-generation diagnostics to reduce the burden of pathogenic microorganisms, mainly in underdeveloped countries. Plasmonics is an optical technology used in disease monitoring, diagnostics, food safety, and biological imaging applications.^152^ At the intersection of analytical chemistry and optics, plasmonic-stemmed modalities can improve the performance of pre-existing platforms by enabling reliable, real-time, susceptible, and label-free detection of analytes while minimizing the need for special equipment.^153,154^ Over the last decades, surface plasmon resonance (SPR)^155−157^ and localized surface plasmon resonance (LSPR)^158,159^ sensors have been created for diagnostic and monitoring applications among many types of label-free technologies.

The most frequently utilized form of the plasmonic biosensor is commonly referred to as SPR. It is widely regarded as the gold standard in optical and plasmonic biosensors.^155^ SPR transfers the signal into a colorimetric sensor via changes in the spectral position and intensity in response to external stimuli. Additionally, SPR can concentrate the incident electromagnetic field in a nanostructure, modify fluorescence emission, and enable ultrasensitive detection using plasmon-enhanced fluorescence.^160^

The latest platform developed by Nawattanapaiboon et al. have achieved 10 copies/μL by employing the LAMP-SPR detection process for the selective detection of methicillin-resistant *Staphylococcus aureus* (MRSA) with very low detection limits.^161^ DNA samples were taken from clinical specimens such as sputum and blood hemoculture to confirm this research. It was subjected to LAMP amplification for DNA segments of the *fem*B and *mec*A genes, respectively, that were 0.18 kbp and 0.23 kbp in size. To detect LAMP amplicons from MRSA, immobilized streptavidin-biotinylated probes on the sensor surface were used to develop a self-assembled monolayer surface (SAMs). Both LAMP amplicons were hybridized with ssDNA probes mounted onto a biofunctionalized surface to identify particular targets in the multiplex DNA array platform. However, this platform can identify MRSA with great sensitivity and without PCR.^161^

Additionally, Nag et al. used bacterial LSPR-bacteriolysis signatures on optical fiber probes for rapid beta-lactam susceptibility testing.^162^ The concept was validated using *P. aeruginosa* and *E. coli* suspended in human urine for prospective medication sensitivity testing for urinary tract infections (UTI). The sensor has tremendous prospects for point-of-care beta-lactam susceptibility testing when utilized in this mode by directly capturing bacteria from suspicious UTI patients. The sensor provides a quick alternative to slow, burdensome drug susceptibility testing methods in hospitals. It is also an alternative to complex analytical apparatus used to identify and quantify selected beta-lactams.^162^

### Electrochemical Detection

Electrical and microelectro-mechanical sensors can be used as alternatives to optical sensing techniques for detecting pathogens. For possible POC diagnosis of AMR, an electrochemical detection technique is currently being investigated. It was designed as a robust and quick point-of-care application that is inexpensively miniaturized. With the advancement in material sciences and fabrication procedures, cumbersome conventional electrodes have been gradually substituted with miniaturized and transferrable electrochemical systems in clinical practice.^163^ To detect pathogens, electrochemical biosensors use conducting and semiconducting materials as the transducer, also referred to as an electrode. When target pathogens bind to electrode-immobilized biorecognition components, the chemical energy associated with this binding is transformed into electrical energy using an electrochemical technique involving the electrode and a pathogen-containing electrolyte solution.^164^

**Figure 7:**
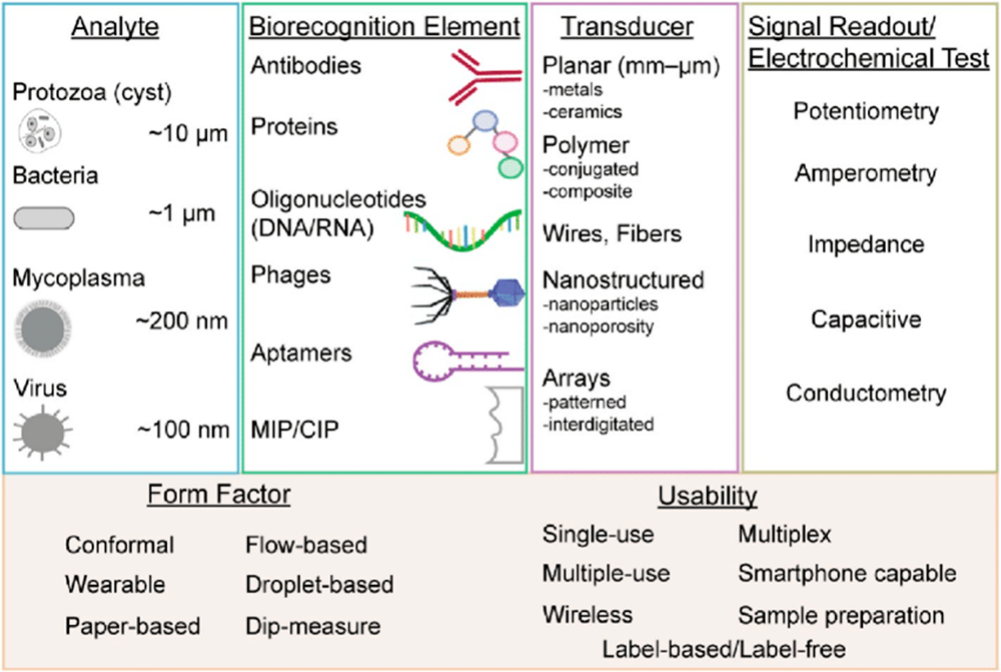
Electrochemical biosensors for pathogen detection: components and measurement formats. Reproduced from Cesewski, E.; Johnson, B. N. Electrochemical Biosensors for Pathogen Detection. *Biosens Bioelectron.***2020**, *159*, 112214 (ref (163)). Copyright 2020, with permission from Elsevier.

Along with the electrochemical analysis, electrochemical detection frequently uses technology such as carbon electrodes and field-effect transistor (FET) biosensors.^165^ Electrochemical sensors offer a broad range of applications in biological sensing because they detect electrochemical changes at electrode interfaces that may be read using voltammetric, potentiometric, or impedimetric techniques.^166^Figure 7 depicts a high-level overview of electrochemical biosensors used in pathogen detection.

Sun et al. developed an easy and inexpensive material for identifying harmful bacteria, particularly antibiotic resistance for human health and safety.^167^ The presence of *E. coli* is regarded as an indication of contamination, and it must be directly linked to human health to be deemed reliable. In this paper, they use biocatalysis of bacterial surfaces to examine the presence of *E. coli* and its relative level of antibiotic resistance. *p*-Benzoquinone is used as a redox mediator in this approach to monitor the bacterial concentration and specifically distinguish *E. coli* from four other common clinical bacteria, namely, *S. aureus*, *Enterococcus faecalis* (*E. faecalis*), *Salmonella pullorum* (*S. pullorum*), and *Streptococcus mutans* (*S. mutans*). A noticeable color shift, taken with a smartphone using a “lightbox” and without the need of any sophisticated apparatus, may be used to determine the number of bacteria in a sample. It may differentiate between *E. coli* at the same concentration from antibiotic-resistant *E. coli*. The use of the CV method accomplished electrochemical detection. In this test, the electrochemical technique was more sensitive in identifying *E. coli* at very low concentrations as 1.0 × 10^3^ CFU/mL within an hour.^167^

Wang et al. have presented a novel approach for detecting ampicillin based on aptamer-based differential pulse voltammetry.^168^ A GCE was modified using double-stranded DNA (dsDNA) carrying an ampicillin aptamer sequence in this technique.^168^ The DPV technique resulted in an outstanding 3.2 × 10^–11^ M detection limit in the real sample.^168^ Zelada-Guillén et al. showed label-free detection and identification of live bacteria in real samples.^169^ It can be performed in a matter of minutes. It is direct, simple, and selective at concentrations as low as 6 CFU/mL in complex matrixces such as milk or 26 CFU/mL in apple juice, with minimal sample preparation required. They chose *E. coli* CECT 675 cells as a model organism as a nonpathogenic surrogate for pathogenic *E. coli* O157:H7 to test the effectiveness of a potentiometric aptamer-based biosensor. SWCNTs are efficient ion-to-electron transducers, and covalently bound aptamers serve as biorecognition components in this biosensor. The selective aptamer targeted contact significantly changes the electrical potential, allowing interspecies selectiveness and direct target detection. As a result, this approach is a highly effective tool for the rapid identification and detection of microorganisms.^169^

## Outlook and Perspective

AMR is a leading global health threat. Most of the commercially available rapid methods for detecting AMR are based on genotypic or phenotypic methods. These traditionally available methods have been confined to a specialist setting due to the cost and size of these devices and the need for on-site expertise. The analysis is the cornerstone of disease diagnosis and management. The development of robust diagnostics that enable decentralized analysis (at home or the point of care) is critical for changing the healthcare paradigm. With the emergence of inexpensive and compact on-site technology capable of detecting AMR at a cost-effective price, a robust and simple-to-use method is in demand by healthcare workers.

A possible answer may lie in the development of point-of-care (POC) testing against key pathogens with a complex resistance profile and high incidence of severe infections. POC is a popular measuring technique in many diseases. The possibility of giving diagnostic results rapidly in nonlaboratory situations provides POC diagnostics as an appealing prospect. These tools will be significant and timely for a physician in delivering a proper antibiotic treatment of their patient’s infections with substantial savings in healthcare costs. For the patients, there will be a reduction in symptoms and consequent improvements in their quality of life. POC medical testing is performed near the patient for quick analysis and diagnosis and therefore enables sample analysis and diagnosis to be transferred directly from central laboratories to the team caring for the patient. It will also reduce the time to obtain pathogen information from days to a few hours. This brings the results of the analysis from the specialist lab to the patients themselves so that they may monitor their own health and could improve the efficacy of prevention and therapy. POC based approaches have been mainly identified in both developed and developing countries. It would also ease AMR surveillance efforts and enable low-resource areas to benefit more fully from rapidly decreasing sequencing costs. Based on the current research, we hypothesize that electrochemical and optical detection holds the most potential for use in portable POC testing.

This review gives a comprehensive review of AMR identification and characterization approaches, ranging from nanomaterials-based detection to conventional methods. We focus on recent detection based on electrochemical and optical methods. It is supposed that this review will provide a foundation for informed decisions and POC parameters for the detection of specific bacteria, which will further be capable of combatting AMR pathogens.
